# Potential of the Angiotensin Receptor Blockers (ARBs) Telmisartan, Irbesartan, and Candesartan for Inhibiting the HMGB1/RAGE Axis in Prevention and Acute Treatment of Stroke

**DOI:** 10.3390/ijms140918899

**Published:** 2013-09-13

**Authors:** Kiyoshi Kikuchi, Salunya Tancharoen, Takashi Ito, Yoko Morimoto-Yamashita, Naoki Miura, Ko-ichi Kawahara, Ikuro Maruyama, Yoshinaka Murai, Eiichiro Tanaka

**Affiliations:** 1 Department of Pharmacology, Faculty of Dentistry, Mahidol University, 6 Yothe Road, Rajthevee, Bangkok 10400, Thailand; E-Mails: kikuchi_kiyoshi@kurume-u.ac.jp (K.K.); salunya.tan@mahidol.ac.th (S.T.); 2 Division of Brain Science, Department of Physiology, Kurume University School of Medicine, 67 Asahi-machi, Kurume 830-0011, Japan; E-Mail: ymurai@med.kurume-u.ac.jp; 3 Department of Neurosurgery, Kurume University School of Medicine, 67 Asahi-machi, Kurume 830-0011, Japan; 4 Department of Systems Biology in Thromboregulation, Kagoshima University Graduate School of Medical and Dental Sciences, 8-35-1 Sakuragaoka, Kagoshima 890-8520, Japan; E-Mails: takashi@m3.kufm.kagoshima-u.ac.jp (T.I.); rinken@m3.kufm.kagoshima-u.ac.jp (I.M.); 5 Department of Restorative Dentistry and Endodontology, Kagoshima University Graduate School of Medical and Dental Sciences, 8-35-1 Sakuragaoka, Kagoshima 890-8544, Japan; E-Mail: yokomaru@dent.kagoshima-u.ac.jp; 6 Laboratory of Diagnostic Imaging, Department of Veterinary Science, Faculty of Agriculture, Kagoshima University, 1-21-24 Korimoto, Kagoshima 890-0065, Japan; E-Mail: nm18@vet.kagoshima-u.ac.jp; 7 Laboratory of Functional Foods, Department of Biomedical Engineering Osaka Institute of Technology, 5-16-1 Omiya, Asahi Ward, Osaka 535-8585, Japan; E-Mail: kawahara@bme.oit.ac.jp

**Keywords:** stroke, telmisartan, irbesartan, candesartan, high mobility group box 1, receptor for advanced glycation end-products

## Abstract

Stroke is a major cause of mortality and disability worldwide. The main cause of stroke is atherosclerosis, and the most common risk factor for atherosclerosis is hypertension. Therefore, antihypertensive treatments are recommended for the prevention of stroke. Three angiotensin receptor blockers (ARBs), telmisartan, irbesartan and candesartan, inhibit the expression of the receptor for advanced glycation end-products (RAGE), which is one of the pleiotropic effects of these drugs. High mobility group box 1 (HMGB1) is the ligand of RAGE, and has been recently identified as a lethal mediator of severe sepsis. HMGB1 is an intracellular protein, which acts as an inflammatory cytokine when released into the extracellular milieu. Extracellular HMGB1 causes multiple organ failure and contributes to the pathogenesis of hypertension, hyperlipidemia, diabetes mellitus, atherosclerosis, thrombosis, and stroke. This is the first review of the literature evaluating the potential of three ARBs for the HMGB1-RAGE axis on stroke therapy, including prevention and acute treatment. This review covers clinical and experimental studies conducted between 1976 and 2013. We propose that ARBs, which inhibit the HMGB1/RAGE axis, may offer a novel option for prevention and acute treatment of stroke. However, additional clinical studies are necessary to verify the efficacy of ARBs.

## 1. Introduction

Stroke was the second most common cause of death worldwide in 2011, resulting in 6.2 million deaths (11.4% of the total) in statistics of the World Health Organization. Stroke is also the second leading cause of death and the leading cause of adult disability in the USA, with an estimated cost of $68.9 billion annually [1,2]. The overall costs of stroke care will account for 6.2% of the total burden of illness by 2020 [2]. The major cause of stroke is atherosclerosis. Three of the most common risk factors for atherosclerosis are hypertension (HT), hyperlipidemia (HL), and diabetes mellitus (DM) [3]. Antihypertensive treatment is recommended for the prevention of stroke because HT is the single most important modifiable risk factor for stroke [4].

Telmisartan, irbesartan, and candesartan are angiotensin receptor blockers (ARBs), which are widely used to treat patients with HT. These ARBs inhibit the expression of the receptor for advanced glycation end-products (RAGE), which is induced by an inflammatory factor, tumor necrosis factor-α (TNF-α), in human endothelial cells [5]. Grossin *et al*. reported that modulating RAGE expression by correcting endothelial dysfunction is achievable by drugs already used for HT or DM treatment, such as ARBs [5]. Therefore, these ARBs may be useful in the prevention of stroke.

## 2. Advanced Glycation End-Products (AGEs) and RAGE

AGEs are modifications of proteins or lipids that become nonenzymatically glycated and oxidized after contact with sugars. Sporadically elevated levels of blood glucose induce the generation of largely irreversible AGEs, which accumulate in tissues and plasma of humans and rodents with normal aging, and their rate of accumulation is greatly increased in DM [6–8].

RAGE is a central cell-surface receptor for AGEs, and is also a multi-ligand receptor, which is a member of the immunoglobulin superfamily of cell-surface molecules [9,10]. RAGE acts as the counter-receptor of integrin, an endothelial adhesion receptor, in leukocytes, and activation of RAGE reduces leukocyte recruitment. Therefore, RAGE propagates cellular dysfunction in several inflammatory disorders [11].

Although the activation of RAGE mediated by its ligands evokes inflammatory cell infiltration and activation of the vessel wall, soluble RAGE (sRAGE), the truncated form spanning the extracellular binding domain of RAGE, shows potent anti-inflammatory effects produced by the action of absorbents for AGEs [12]. Soluble RAGE, which potentially counteracts AGEs, consists of several forms, including endogenous secretory RAGE (esRAGE; a splice variant of RAGE) and cleaved-type sRAGE, which is derived from cell-surface RAGE [13]. A review by Schmidt *et al*. demonstrated that the binding of AGEs to RAGE is a critical step in a mechanism for chronic vascular dysfunction in diabetic vasculopathy and atherosclerosis [14].

## 3. High Mobility Group Box 1 (HMGB1)

HMGB1, a predominantly nuclear protein, is expressed in many eukaryotic cells with a highly conserved sequence among species [15]. HMGB1 acts as an intracellular regulator of transcription, such as p53 and nuclear factor-κB (NF-κB), and plays a crucial role in the maintenance of DNA functions [16]. RAGE is able to bind some ligands (e.g., HMGB1 and AGEs) and is implicated in many HMGB1 effects [17]. HMGB1 is released from various cells (e.g., macrophages/monocytes, endothelial cells, and pituicytes) when these cells are stimulated by lipopolysaccharide or TNF-α. HMGB1 acts as a proinflammatory cytokine through RAGE and toll-like receptors 2 and 4, and it is also released from necrotic cells [18–24]. HMGB1 is a member of the “alarmin” family, a group of endogenous factors that act from the extracellular space after release, and also activate the inflammatory response through the binding of membrane receptors [25,26]. HMGB1 has been identified as a lethal mediator of severe sepsis. HMGB1 levels are markedly increased during severe sepsis in humans and mice. Administration of neutralizing antibodies that are specific for HMGB1 reduces the lethality of mice with sepsis [23]. In addition, HMGB1 contributes to the pathogenesis of disorders in various organs, such as the heart, liver, lungs, gut, pancreas, joints, spine, and periodontium, and is implicated in graft rejection in transplantation [22,23,27–38]. Furthermore, inactivation of HMGB1 using an anti-HMGB1 monoclonal antibody (McAb) or short hairpin RNA-mediated HMGB1 has already been shown to be effective in various animal models of cancer, rheumatoid arthritis, myocardial infarction, hepatic ischemia, acute pancreatitis, hemorrhagic shock, and sepsis [23,39–44].

However, anti-HMGB1 and short hairpin RNA-mediated HMGB1 are not currently used clinically. Therefore, clinically approved drugs, such as telmisartan, irbesartan, and candesartan, which inhibit the HMGB1/RAGE axis, may be useful in the prevention and treatment of various diseases.

## 4. Experimental Studies of ARBs

*In vitro* studies using neuron–astrocyte co-cultures have demonstrated protective effects of telmisartan on ischemic neuronal injury [45]. Oxygen-glucose deprivation (OGD) is widely used as an *in vitro* ischemic model [46]. Telmisartan attenuates OGD-induced cellular damage, and suppresses OGD-induced extracellular release of glutamate, production of reactive oxygen species (ROS), and generation of nitric oxide (NO) [45].

*In vivo* studies have demonstrated protective effects of telmisartan, irbesartan, and candesartan on neuronal injury. Stroke-prone spontaneously hypertensive rats (SHRSPs), which developed from normotensive Wistar Kyoto rats, have proven useful for the study of the pathogenesis of stroke and for the testing of prophylactic anti-stroke compounds [47,48]. SHRSPs develop severe hypertension with age and die from ischemic stroke or hemorrhagic stroke in greater than 80% of the animals [47]. Telmisartan reduces the incidence of stroke, prolongs survival, and improves neurological outcome in SHRSPs [49]. Irbesartan also increases the survival rate in SHRSPs fed a high-salt and low-protein diet, and ameliorates the appearance of stroke symptoms, showing an association with the prevention of microscopic lesions [50]. Candesartan reduces the incidence of stroke in SHRSPs [51]. These findings demonstrate that telmisartan, irbesartan, and candesartan prevent stroke in SHRSPs.

Middle cerebral artery occlusion (MCAO) is widely used as an animal model of ischemic stroke. Tyrosine-related kinase B (TrkB) is the receptor of brain-derived neurotrophic factor (BDNF) [52]. BDNF acts on certain neurons of the central and peripheral nervous systems to support the survival of existing neurons, and encourage the growth and differentiation of new neurons and synapses [53,54]. Telmisartan improves neurological outcome, reduces infarct size and TNF-α levels, and induces expression of the TrkB receptor and neuronal survival in a rat MCAO model [49]. Irbesartan also improves neurological outcome, reduces infarct size, decreases the number of apoptotic cells in the peri-infarct cortex, and attenuates the invasion of activated microglia and macrophages in the peri-infarct cortex in the rat MCAO model [55]. Furthermore, irbesartan decreases TNF-α levels, and inhibits the monocyte chemoattractant protein-1 (MCP-1)/C-C chemokine receptor 2 (CCR2) signaling pathway in the rat MCAO model [56]. Candesartan reduces infarct size, improves neurological outcome, increases cerebral blood flow, and stimulates the neurotrophin BNDF/TrkB system in the rat MCAO model [57,58]. Inhibition of metalloproteinase (MMP)-2 and MMP-9 reduces neuronal and glial apoptosis [59]. Furthermore, Guan *et al.* reported that MMP-2, MMP-9, and vascular endothelial growth factor (VEGF) are significantly increased by MCAO, but candesartan fails to reduce MMP-2, MMP-9, and VEGF in the rat MCAO model [60]. These findings show that telmisartan, irbesartan, and candesartan reduce infarct size and improve neurological outcome in ischemic stroke model rats.

Many experimental studies on stroke model animals have demonstrated that telmisartan, irbesartan, and candesartan have protective effects on the structure of neurons and vessels (Table 1), and fulfill many Stroke Therapy Academic Industry Roundtable (STAIR) criteria [61]. However, data from *in vivo* and *in vitro* studies indicate that it is not clear whether inhibition of the HMGB1/RAGE axis directly contributes to the prevention and treatment of stroke.

## 5. Clinical Studies of ARBs

The three ARBs, telmisartan, irbesartan, and candesartan, were effective in several double-blind clinical studies for stroke (Table 2). These ARBs were administered by various methods, such as oral, intravenous, interacisternal or intraperitoneal in animal studies, whereas, these ARBs were administered by oral in clinical studies. Therefore, some factors, such as BBB permeability of the drug, concentration of the drug in the certain brain tissue and drug metabolism may contribute to the discrepancy between animal studies and clinical studies with regards to prevention and treatment outcomes.

### 5.1. Telmisartan

The results of three recent clinical studies of telmisartan, including the Prevention Regimen For Effectively avoiding Second Strokes (PRoFESS) [62], Ongoing Telmisartan Alone and in Combination with Ramipril Global Endpoint Trial (ONTARGET) [63], and the Telmisartan Randomized Assessment Study in ACE-intolerant Subjects with Cardiovascular Disease (TRANSCEND) [64] have suggested a modest neurovascular protective effect of telmisartan against stroke events. The PRoFESS study compared placebo with telmisartan in 20,332 patients who recently had an ischemic stroke [62]. Therapy with telmisartan (80 mg per day) initiated soon after an ischemic stroke (the median interval was 15 days) and continued for 2.5 years, did not significantly lower the rate of recurrent stroke, but there was a trend for the reduction of recurrent stroke (*p* = 0.23). The PRoFESS study group suggested that the duration of the study may have been too short, which may have contributed to a lack of significant benefit associated with telmisartan. The ONTARGET study compared an angiotensin-converting enzyme (ACE) inhibitor, ramipril (10 mg per day), telmisartan (80 mg per day), and a combination of both drugs in 25,611 patients with vascular disease or high-risk DM over a median follow-up period of 56 months [63]. Ramipril, telmisartan, and combination therapy proved to be equivalent with regard to the prevalence of recurrent stroke. Furthermore, the TRANSCEND study compared placebo with telmisartan (80 mg per day) in 5926 patients intolerant to ACE inhibitors over a median follow-up period of 56 months. The TRANSCEND study found that the incidence of stroke was reduced by 17% in the telmisartan group, but significant differences were not noted (*p* = 0.136) [64]. Incidentally, these studies on telmisartan such as PRoFESS, ONTARGET, and TRANSCEND, were supported by Boehringer Ingelheim.

### 5.2. Irbesartan

Clinical studies on prevention and acute treatment of stroke have been performed for irbesartan. The Atrial Fibrillation Clopidogrel Trial with Irbesartan for Prevention of Vascular Events (ACTIVE I) study compared placebo with irbesartan once daily (at a dose of 150 mg per day for 2 weeks, and 300 mg per day thereafter) in 9016 patients with atrial fibrillation over a median follow-up period of 4.1 years [65]. Irbesartan significantly reduced the incidence of stroke, transient ischemic attack, and non-central nervous system embolism compared with placebo in this study (*p* = 0.02). However, irbesartan did not show the significant reduction in incidence of stroke compared with placebo in this study (*p* = 0.20). This ACTIVE I study was supported by Pfizer.

Furthermore, irbesartan (150 mg per day) was compared with placebo in 43 patients in a clinical study of early treatment of acute ischemic stroke [66]. There were no significant differences between these two groups in neurological outcome (National Institutes of Health Stroke Scale [NIHSS] score) after 30 days (*p* = 0.066). The sample size in this study may have been too small, which may have contributed to the lack of significant benefit associated with irbesartan. This study was supported by Bristol-Myers Squibb and Sanofi-Aventis.

### 5.3. Candesartan

Clinical studies of prevention and treatment of stroke have also been performed for candesartan. The efficacy of candesartan in preventing stroke was tested in the Study on Cognition and Prognosis in the Elderly (SCOPE). The SCOPE study compared placebo with candesartan (8–16 mg per day) in 4964 patients [67]. There were significant reductions in nonfatal stroke (*p* = 0.04) and a trend for a reduction in all types of stroke (*p* = 0.06) in the candesartan group. Incidentally, this SCOPE study was supported by AstraZeneca. Although candesartan also prevented stroke in two clinical studies, Candesartan Antihypertensive Survival Evaluation in Japan (CASE-J) [68] and Efficacy of Candesartan on Outcome in Saitama Trial (E-COST) [69], these studies were not double-blind tests. Furthermore, the efficacy of candesartan for preventing stroke and improving neurological outcome (Barthel index) was tested in the Scandinavian Candesartan Acute Stroke Trial (SCAST). The SCAST study compared placebo with candesartan (doses increasing from 4 mg on day 1 to 16 mg on days 3 to 7) in 2029 patients with acute stroke (ischemic or hemorrhagic) [70]. There were no significant differences among both groups in the incidence of stroke (*p* = 0.38) and neurological outcome (*p* = 0.47) after six months. However, in patients within 6 h of symptom onset, there was a trend in preventing the incidence of stroke and improving clinical outcome in the candesartan group (*p* = 0.08). This SCAST study was supported by AstraZeneca.

Recently, the comparative effectiveness of ARBs for preventing stroke, heart failure, or acute myocardial infarction, such as telmisartan, irbesartan, candesartan, losartan, or valsartan, was investigated in 54,186 patients with DM in a population-based randomized cohort study [71]. The study results suggested that telmisartan and valsartan are associated with a lower risk of admission to hospital for stroke, heart failure, or acute myocardial infarction in older patients with DM [71]. Currently, comparing the efficiency in reduction of the incidence of stroke events and improvement in neurological outcome may not be possible for these ARBs in randomized, controlled clinical studies. However, in major clinical studies, the application of ARBs shows a trend towards the reduction of recurrent stroke.

## 6. HMGB1/RAGE and Risk Factors of Stroke

HMGB1 acts as a proinflammatory cytokine, and may play a role in the progression of risk factors of stroke, such as HT, HL, DM, atherosclerosis, and thrombosis. Inhibition of the HMGB1/RAGE axis may be an effective treatment for these risk factors of stroke.

### 6.1. Hypertension

HMGB1 and RAGE contribute to the pathogenesis of HT. A hypertensive challenge has an early and sustained effect on RAGE upregulation in brain vessels of the cortex and hippocampus in mice [72]. Plasma sRAGE levels are decreased in patients with essential HT [73]. Nakamura *et al*. reported that in patients with essential HT, plasma sRAGE levels are associated with body mass index, waist circumference, and alanine aminotransferase, gamma-glutamyltranspeptidase, 24-hour creatinine clearance, B-type natriuretic peptide, and TNF-α levels [74]. Telmisartan decreases serum sRAGE levels in patients with essential HT [75]. In addition, Nakamura *et al*. reported that telmisartan significantly reduced serum HMGB1 levels compared with enalapril (ACE inhibitor) in autosomal dominant polycystic kidney disease patients with HT [76]. These findings show that RAGE may contribute to the pathogenesis of HT. It is possible that the hypertensive challenge may upregulate RAGE in brain vessels in human, and that telmisartan may inhibit serum HMGB1 levels in patients with HT. However, it is still unclear whether telmisartan inhibits upregulation of RAGE in patients with HT, and whether it inhibits relative activation of RAGE in patients with HT. Because telmisartan decreases serum sRAGE and HMGB1 levels in patients with HT, either inhibition or augmentation of the HMGB1/RAGE axis could occur in patients with HT.

### 6.2. Hyperlipidemia

HMGB1 and RAGE also contribute to the pathogenesis of HL. Cell culture experiments using monocyte cell culture (U937) have demonstrated relocation of HMGB1 from the nucleus to the cytoplasm under hyperlipidemic stress [77]. A high level of HMGB1 in serum and the cytosol of lung epithelial cells are positively correlated with the upregulation of RAGE in lung tissue from hyperlipidemic golden Syrian hamsters [77]. Santilli *et al*. reported that sRAGE in plasma was significantly lower in hypercholesterolemic patients than in normocholesterolemic patients [78]. Therefore, some experimental and clinical studies have demonstrated that HMGB1 and RAGE may contribute to the pathogenesis of HL. In human, it is possible that the hyperlipidemic stress may increase in HMGB1 level in serum and upregulate RAGE in tissue. At least, RAGE may be upregulated as a result of HT and HL, rather than being the cause of HT and HL.

### 6.3. Diabetes Mellitus

HMGB1 and RAGE are thought to play a role in the progression of DM [79]. Hyperglycemia increases the expression of HMGB1 and RAGE in human aortic endothelial cells [80]. Moreover, earlier and enhanced accumulation of AGEs in DM has been linked to the pathogenesis of vascular and inflammatory cell complications that typify DM [6–8]. Ichikawa *et al*. reported that AGEs induce expression of tissue factor in human monocyte-like U937 cells and in monocytes from diabetic patients, and that AGEs may promote thrombosis and the development of atherosclerosis by inducing expression of tissue factor in monocytes in patients with DM [81]. Furthermore, *in vitro* experiments have shown that RAGE binding to AGEs leads to changes in endothelial cells and pericytes, which are similar to the characteristics known as diabetic microangiopathy [82]. Activation of RAGE induces elevation in generation of superoxide by mononuclear phagocytes in patients with DM [83]. These *in vitro* studies have demonstrated that AGEs and RAGE may contribute to the pathogenesis of diabetic microangiopathy in DM.

When the expression of RAGE has been interrupted by using small interfering RNA (siRNA) in diabetic human monocytes, a reduction in RAGE significantly inhibits the expression of CD36 and the production of ROS [84]. This suggests a positive interaction between the expression of RAGE and the expression of CD36 and generation of ROS in monocytes. Vascular smooth muscle cells (VSMCs) derived from insulin-resistant and diabetic db/db mice display an increased expression of RAGE and migration capability compared with VSMCs derived from control db/+ mice. The application of RAGE antibodies interferes with enhanced migration capability in db/db cells [85]. Based on these results, inhibition of RAGE may reduce oxidative stress and migration of VSMCs in DM.

RAGE is expressed in many cell types, including endothelial cells, monocytes, smooth muscle cells, and fibroblasts, at low levels in healthy adult animals, but at significantly higher levels in DM [86–88]. Lewis *et al*. reported an increase in expression of RAGE, vascular cell adhesion molecule-1 (VCAM-1), VEGF, and connective tissue growth factor in diabetic ApoE(−/−) mice [89]. RAGE-induced cytosolic production of ROS facilitates the production of mitochondrial superoxide in hyperglycemic environments. A deficiency in RAGE prevents diabetes-induced increases in renal mitochondrial superoxide and renal cortical apoptosis in diabetic RAGE(−/−) mice [90]. These *in vivo* studies indicate that RAGE may contribute to an increase in oxidative stress and apoptosis in the DM model mouse kidney.

Adipocyte hypertrophy induced by overexpression of RAGE is associated with suppression of glucose transporter type 4 and adiponectin mRNA expression, attenuated insulin-stimulated glucose uptake, and insulin-stimulated signaling [91]. RAGE (−/−) mice have significantly lower body weight, higher levels of adiponectin in serum, and higher sensitivity of insulin than wild-type mice. Furthermore, diabetic RAGE transgenic mice that overexpress RAGE in vascular cells exhibit exacerbation of nephropathy and retinopathy, and these are prevented by the inhibition of formation of AGEs [82]. In contrast to RAGE-overexpressing mice, diabetic RAGE knockout mice show marked improvement of nephropathy. These *in vivo* studies suggest that inhibition of RAGE may contribute to the pathogenesis of nephropathy and retinopathy in the DM model mouse.

The anti-RAGE McAb, but not control Ab, prevents upregulation of transforming growth factor-β, development of submesothelial fibrosis, and fibronectin accumulation in the peritoneum of diabetic rats [92]. Candesartan significantly reduces expression of connective tissue growth factor and effectively suppresses the development of fibrotic deposition in diabetic rats [93]. Furthermore, candesartan reduces accumulation of AGEs and subsequent albuminuria by downregulating the nicotinamide adenine dinucleotide phosphate oxidase p47phox component and by inducing NO synthase expression, as well as by attenuating expression of RAGE in type 2 diabetic KK/Ta mouse kidneys [94]. These findings indicate that candesartan may have therapeutic effects in animal models of DM with a reduction in AGEs and RAGE.

Many clinical studies have also demonstrated a relationship between DM and the HMGB1/RAGE axis. Levels of AGEs in serum and in plaques of patients with type 2 DM are higher than those in non-diabetics [95–97]. Cipollone *et al*. reported that overexpression of RAGE was associated with an enhanced inflammatory reaction and cyclooxygenase-2/microsomal prostaglandin E synthase-1 expression in diabetic plaque macrophages [98]. This effect may contribute to plaque destabilization by inducing expression of MMP [98]. Despite an increase in expression of RAGE, expression of esRAGE is downregulated in the plaques of diabetic patients, and sRAGE and esRAGE levels in serum or plasma are also reduced compared with those in controls [99–103]. Univariate regression analysis showed that serum sRAGE levels are positively correlated with inflammatory markers, such as MCP-1 and TNF-α, in patients with type 2 DM [104]. In patients with diabetic microangiopathy, mean sRAGE levels in serum are significantly decreased compared with those in diabetic patients without microvascular complications [105]. Furthermore, the level of circulating esRAGE is significantly lower in type 1 diabetic patients than in non-diabetic subjects, and is inversely associated with the severity of some diabetic vascular complications [106]. Circulating serum AGE and sRAGE levels are associated with the severity of nephropathy in type 2 DM patients [107]. Niu *et al*. reported convincing evidence regarding the association of the RAGE gene 1704T allele with an increase in risk of DM in a meta-analysis [108]. HMGB1 concentrations in serum are elevated in non-diabetic hyperglycemic patients and diabetic patients [109–111]. In a 12-year follow-up study, Nin *et al*. reported that higher plasma levels of HMGB1 were independently associated with a higher risk of all-cause mortality, and to a lesser extent, with a higher incidence of cardiovascular disease in patients with type 1 DM [112]. These clinical studies suggest that the HMGB1/RAGE axis may contribute to the pathogenesis of DM. Pharmacological intervention with AGEs, RAGE, and HMGB1 might provide a potential therapy for DM.

### 6.4. Atherosclerosis

HMGB1 and RAGE also contribute to the pathogenesis of atherosclerosis. Hu *et al*. reported that AGE-induced autophagy of VSMCs contributes to the process of AGE-induced proliferation of rat VSMCs, which is related to atherosclerosis in DM [113]. AGEs increase cell migration and proliferation in cultured rat VSMCs, and these effects interfere with anti-RAGE antibody [114]. Overexpression of RAGE induces MCP-1 expression in the rat VSMC line [115]. Telmisartan inhibits AGE-elicited cell injury by suppressing expression of RAGE via peroxisome proliferator-activated receptor-γ activation in human cultured microvascular endothelial cells [116]. Candesartan decreases TNFα induced RAGE expression at the mRNA and protein level along with a decrease in activity of NF-κB and expression of inflammatory mediators, such as VCAM-1 in human endothelial cells. [117]. In rat vascular adventitial fibroblasts, AGEs upregulate the expression of RAGE, and significantly increase the migration capability and the release of inflammatory mediators, such as MCP-1, interleukin-6, and VCAM-1 [118]. AGEs also upregulate NF-κB transcriptional activity and I kappaB alpha phosphorylation [118]. The application of candesartan, as well as anti-RAGE neutralizing antibody, significantly attenuates AGE-induced increase in RAGE, migration capability, and release of inflammatory mediators in rat vascular adventitial fibroblasts. These results suggest that telmisartan and candesartan may have anti-atherosclerotic effects.

Wang *et al*. showed in cell and animal studies that N(ɛ)-carboxymethyl-lysine (CML), a major immunogen of AGE and the RAGE axis, may first initiate apoptosis of macrophages in atherosclerotic lesions and then induce the bone morphogenetic protein 2-core-binding factor α1-alkaline phosphatase-calcification cascade in a high-lipid, apoptosis-coexisting environment. CML is known as a major immunogen of AGE. Wang *et al*. showed that CML and RAGE were present in aortic lesions in diabetic apoE(−/−) mice [119]. Moreover, CML augments apoptosis of macrophages in a high-lipid environment in mice. CML also accelerates calcification of rat aortic VSMCs under high-lipid, apoptosis-coexisting conditions [119]. In response to carotid artery ligation in wild type mice, expression of RAGE is increased in the tissue of carotid arteries in a time-dependent manner. There is marked neointimal formation in the proximal area of ligation of carotid arteries in wild type mice, but this neointimal formation is attenuated in RAGE(−/−) mice [120]. Diabetic RAGE(−/−)/apoE(−/−) double knockout mice have significantly reduced atherosclerotic plaque areas compared with diabetic apoE(−/−) mice [121]. These effects on the vasculature produced by a reduction or absence of RAGE are associated with attenuation of leukocyte recruitment and decreased expression of proinflammatory mediators, including the NF-κB subunit RelA (p65), VCAM-1, and MCP-1. These effects on the vasculature are also associated with reduced oxidative stress because reduction of RAGE reduces expression of various nicotinamide adenine dinucleotide phosphate oxidase subunits, such as gp91phox, p47phox, and Rac1 [121]. Furthermore, atherosclerotic lesions and the average necrotic core area in the brachiocephalic arteries of old RAGE(−/−)/apoE(−/−) mice are significantly smaller than those in old RAGE(+/+)/apoE(−/−) mice [122]. Accumulation of HMGB1 is also significantly reduced in the atherosclerotic lesions of RAGE(−/−)/apoE(−/−) mice. These results indicate that reduction of RAGE inhibits expression of proinflammatory mediators and oxidative stress, and then attenuates changes in the vasculature with atherosclerosis. Therefore, it is possible that the HMGB1/RAGE axis contributes to the pathogenesis of atherosclerosis.

Blockade of RAGE suppresses vascular hyperpermeability in rodent models of diabetic complications [123]. Furthermore, blockade of RAGE also suppresses atherosclerotic lesions in diabetic apoE(−/−) mice [123]. Blockade of RAGE also prevents neointimal formation in atherosclerotic lesions of hyperglycemic fatty Zucker rats subjected to balloon de-endothelialization injury of the carotid artery [123]. Similar to humans, atherosclerotic lesions express HMGB1 in diabetic apoE(−/−) mice that are fed a high-fat diet [124]. HMGB1 leads to macrophage migration, expression of proinflammatory mediators, and accumulation of immune and smooth muscle cells in diabetic apoE(−/−) mice [124]. However, treatment with anti-HMGB1 McAb attenuates atherosclerosis, macrophage accumulation in atherosclerotic lesions, and expression of VCAM-1, MCP-1, TNF-α, and interleukin-1β in diabetic apoE(−/−) mice [124]. Furthermore, treatment with candesartan or anti-HMGB1 McAb leads to antiatherosclerotic effects in diabetic apoE(−/−) mice [125]. Candesartan is more effective at reducing accumulation of macrophages and abundance of collagen I in atherosclerotic plaques than rosuvastatin (antilipid drug) in diabetic apoE(−/−) mice [125]. Therefore, these studies demonstrate that inhibition of HMGB1 or RAGE may attenuate changes in vasculature with atherosclerosis.

In human atherosclerotic lesions, HMGB1 is expressed in endothelial cells, some intimal smooth muscle cells, and macrophages [126]. Inoue *et al*. reported that HMGB1 produced by the activation of VSMCs might contribute to the progression and vulnerability of human atherosclerotic lesions toward rupture [127]. Furthermore, Koyama *et al*. reported that the levels of esRAGE in human plasma are associated with components of atherosclerosis, and that esRAGE is a novel and potential protective factor for atherosclerosis [128]. Serum esRAGE levels are an independent risk factor for the progression of carotid atherosclerosis in type 1 DM [129]. Therefore, the HMGB1/RAGE axis may contribute to the pathogenesis of atherosclerosis, and inhibition of the HMGB1/RAGE axis may offer a novel option for treatment of atherosclerosis.

Furthermore, HMGB1 contributes to the pathogenesis of thrombosis. Ito *et al*. reported that HMGB1 inhibits the anticoagulant protein C pathway *via* the thrombin-thrombomodulin complex, and stimulates expression of tissue factor in monocytes *in vitro*, and that HMGB1 promotes development of microvascular thrombosis in rats [130]. It is possible that RAGE may be upregulated as a result of HT and HL, rather than being the cause of HT and HL in human. However, based on these findings, the HMGB1/RAGE axis could be a potential therapeutic target of risk factors of stroke, including HT, HL, DM, atherosclerosis, and thrombosis.

## 7. HMGB1/RAGE in Acute Stroke

*In vitro* and *in vivo* studies have clearly demonstrated the relationship between HMGB1/RAGE axis and acute stroke. In PC12 cells, levels of RAGE are increased after OGD culture [131]. Notably, blockade of RAGE with a selective RAGE antibody *in vitro* reduces the cytotoxicity caused by OGD. Oxygen deprivation triggers upregulation of the expression of the early growth response-1 gene and protein by activation of RAGE in murine aortic endothelial cells [132]. Rapid activation of the early growth response-1 gene and protein in hypoxia also upregulates fundamental inflammatory and prothrombotic stress genes.

Kikuchi *et al*. reported that HMGB1 is released into the extracellular space in the rat MCAO model and in PC12 cells exposed to OGD, and that HMGB1 induces apoptosis in PC12 cells [133,134]. Elevation of HMGB1 levels in the mouse brain induces memory abnormalities [135]. Moreover, Pedrazzi *et al*. reported that HMGB1 might inhibit glutamate uptake in adult mouse glia [136]. RAGE levels are elevated in the mouse brain after experimental stroke and systemic hypoxia, and increased expression of RAGE is mediated by hypoxia-inducible factor-1 in neurons after hypoxia or ischemia [137]. In MCAO rats, RAGE levels are high in the ischemic hemisphere relative to the non-ischemic hemisphere, and the expression of RAGE is primarily located in the penumbra of the ischemic hemisphere [131]. These results indicate that the release of HMGB1 and expression of RAGE in neurons may be increased in the stroke model.

The number of surviving neurons in the hippocampal CA1 region is significantly higher in RAGE(−/−) mice than in wild-type mice after bilateral common carotid artery occlusion [138]. The size of MCAO-induced infarct areas in RAGE-knockout mice is small compared with that in wild-type mice [139]. Furthermore, activation of macrophages is decreased in RAGE-knockout mice [139]. RAGE might contribute to delayed neuronal death after global cerebral ischemia mediated by enhancement of vascular injury and deleterious glia-mediated inflammation. The reduction of RAGE may reduce the infarct area and inflammation in stroke model mice.

Anti-HMGB1 McAb- or short hairpin RNA-mediated HMGB1 protects against brain injury by improving neurological outcome and infarction size in the rat MCAO model [140,141]. Furthermore, anti-HMGB1 McAb inhibits the development of brain edema through the protection of the blood–brain barrier and efficient clearance of circulating HMGB1 [142]. Treatment with telmisartan and anti-HMGB1 McAb- or short hairpin RNA-mediated HMGB1 has neurovascular protective effects [140–143]. Telmisartan inhibits expression of HMGB1 in macrophages/microglia via a peroxisome proliferator-activated receptor-γ-dependent mechanism in the mouse MCAO model [143]. These findings suggest that inhibition of HMGB1 may reduce the infarct area in stroke model animals.

Furthermore, clinical and experimental studies have demonstrated a relationship between the HMGB1/RAGE axis and patients with acute stroke. HMGB1 is significantly elevated in the serum of patients with acute ischemic stroke compared with healthy volunteers [28]. Recently, Huanq *et al.* measured serum HMGB1 levels in 338 acute ischemic stroke patients, and reported that serum HMGB1 levels were an independent predictor of 1-year neurological outcome [144]. Similar results have been reported in patients with hemorrhagic stroke, as well as ischemic stroke. Compared with controls, patients with hemorrhagic stroke have markedly elevated serum HMGB1 levels, which are significantly correlated with IL-6 and TNF-α levels, and neurological outcome at 3 months [145]. Stroke patients express elevated RAGE levels in the ischemic hemisphere relative to the non-ischemic hemisphere [131]. Yokota *et al*. reported that low plasma sRAGE levels are associated with severe leukoaraiosis in acute stroke patients [146]. Moreover, Zimmerman *et al*. reported that AGEs might contribute to the severity of stroke patients with DM and other conditions characterized by AGE accumulation [147]. Therefore, these findings indicate that the HMGB1/RAGE axis may contribute to the pathogenesis of stroke. Neuronal cell death associated with cerebral ischemia is due to a lack of oxygen and glucose, and may involve the loss of ATP, excitotoxicity of glutamate, oxidative stress, reduction of neurotrophic support, and other metabolic stresses [148]. In addition to these factors, HMGB1 and RAGE may also contribute to exacerbation of cerebral ischemia. Therefore, the HMGB1/RAGE axis may provide a target for therapy in stroke.

## 8. Conclusions

Stroke is a frequent and severe neurovascular disorder. Therefore, prevention and treatment of stroke are crucial issues in humans. To prevent stroke, focus should be placed on reduction of risk factors, such as HT, HL, DM, atherosclerosis, and thrombosis. To reduce the risk of stroke, three ARBs, telmisartan, irbesartan, and candesartan, may be candidates for providing good outcomes. A complementary strategy is optimal for management of the risk factors for stroke. Therefore, choosing antihypertensive drugs for the prevention of stroke is important. Furthermore, various drugs other than thrombolytic drugs should be urgently initiated when stroke occurs.

**Figure 1:**
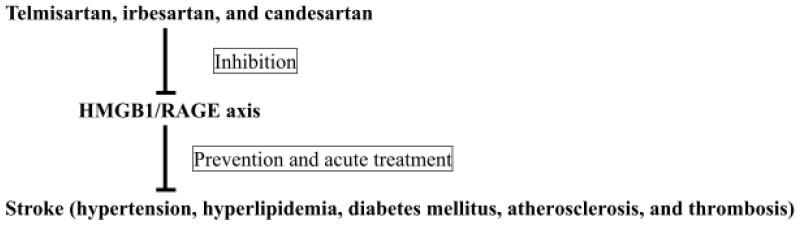
Potential of telmisartan, irbesartan, and candesartan for inhibiting the HMGB1/RAGE axis in prevention and acute treatment of stroke.

Currently, there is no evidence in clinical stroke research that ARBs are effective, even whether ARBs inhibit the HMGB1/RAGE axis. In patients who are currently taking the ARBs telmisartan, irbesartan, and candesartan, it is unclear whether the effect of inhibition of the HMGB1/RAGE axis directly contributes to stroke therapy. However, the extracellular release of HMGB1 appears to be common to the etiology and progression of multiple diseases leading to stroke, and may act in a multi-stage process in stroke. Therefore, the HMGB1/RAGE axis could be considered as a candidate molecular target for the treatment of stroke. Inhibition of the HMGB1/RAGE axis may prove to be a novel therapeutic strategy for treating stroke. Therefore, the inhibitory effect of ARBs on the HMGB1/RAGE axis may provide potential therapeutic effects in patients who require prevention and acute treatment of stroke (Figure 1). Telmisartan may be the most effective in terms of inhibition of RAGE via peroxisome proliferator-activated receptor-activation [5,149]. The choice of the optimal ARB for stroke therapy is difficult because there are few clinical studies in which ARBs have been directly compared regarding their neurovascular protective effects on stroke. Randomized, controlled clinical studies with large sample sizes should be performed. Studies using SHRSPs to compare the effects of all ARBs may be the most plausible means of comparison of the neurovascular protective effects of each ARB against stroke. New scientific strategies should continue to be explored to provide optimal care to stroke patients.

## Figures and Tables

**Table 1:** Stroke Therapy Academic Industry Roundtable (STAIR) quality of telmisartan, irbesartan, and candesartan.

Item	Description	Telmisartan	Irbesartan	Candesartan
Laboratory setting	Focal model tested in two or more laboratories	√	√	√
Animal species	Focal model tested in two or more species	√	√	√
Health of animals	Focal model tested in old or diseased animals (e.g., diabetic, hypertensive, aged, and hyperglycemic)	√	√	√
Sex of animals	Focal model tested in male and female animals	×	×	×
Reperfusion	Tested in temporary and permanent models of focal ischemia	√	√	√
Time window	Drug administered at least 1 h after occlusion in focal model	×	×	√
Dose response	Drug administered using at least two doses in focal model	√	√	√
Route of delivery	Tested using a feasible mode of delivery (e.g., not intracisternal or intraventricular, cortical transplant or graft only)	×	×	×
Endpoint	Behavioral and histological outcomes measured	√	√	√
Long-term effect	Outcome measured at 4 or more weeks after occlusion in focal models	√	×	×
Total		7	6	7

The number of STAIR criteria met by the drug (maximum: 10) are listed.

**Table 2:** Major clinical studies of telmisartan, irbesartan, and candesartan in stroke.

Drugs	Effect for stroke	Sample size	*p* value	Study name	Reference
Telmisartan	Prevention	20,332	0.23	PRoFESS	[62]
	Prevention	25,611	–	ONTARGET	[63]
	Prevention	5926	0.136	TRANSCEND	[64]

Irbesartan	Prevention	9016	0.20	ACTIVE I	[65]
	Acute treatment	43	0.066	–	[66]

Candesartan	Prevention	4964	0.06	SCOPE	[67]
	Prevention	4728	0.198	CASE-J	[68]
	Prevention	2048	–	E-COST	[69]
	Prevention, acute treatment	2029	0.38, 0.47	SCAST	[70]

## Abbreviations

Ab: antibody
ACE: angiotensin-converting enzyme
ACTIVE I: Atrial Fibrillation Clopidogrel Trial with Irbesartan for Prevention of Vascular Events
AGEs: advanced glycation end-products
ALP: alkaline phosphatase
ALT: alanine aminotransferase
ApoE(−/−): apolipoprotein E-deficient
ARB: angiotensin receptor blocker
BBB: blood–brain barrier
BDNF: brain-derived neurotrophic factor
BMI: body mass index
BMP-2: bone morphogenetic protein 2
BNP: B-type natriuretic peptide
CAD: coronary artery disease
CASE-J: Candesartan Antihypertensive Survival Evaluation in Japan
cbfα1: core-binding factor α1
CCr: creatinine clearance
CCR2: C-C chemokine receptor 2
CML: *N*(ɛ)-carboxymethyl-lysine
COX-2: cyclooxygenase-2
DM: diabetes mellitus
EC: endothelial cells
E-COST: Efficacy of Candesartan on Outcome in Saitama Trial
EGR-1: early growth response-1
esRAGE: endogenous secretory RAGE
GGT: gamma-glutamyltranspeptidase
HL: hyperlipidemia
HMGB1: high mobility group box 1
HT: hypertension
IL: interleukin
iNOS: induced nitric oxide synthase
MAPK: mitogen-activated protein kinase
McAb: mono-clonal antibody
MCAO: occlusion of the middle cerebral artery
MCP-1: monocyte chemoattractant protein-1
MMP: metalloproteinase
mPGES-1: microsomal prostaglandin E synthase-1
NF-κB: nuclear factor-κB
NO: nitric oxide
OGD: oxygen-glucose deprivation
ONTARGET: Ongoing Telmisartan Alone and in Combination with Ramipril Global Endpoint Trial
PRoFESS: Prevention Regimen for Effectively Avoiding Second Strokes
RAGE: receptor for advanced glycation end-products
ROS: reactive oxygen species
SCAST: Scandinavian Candesartan Acute Stroke Trial
SCOPE: Study on Cognition and Prognosis in the Elderly
SHRSP: stroke-prone spontaneously hypertensive rat
si: small interfering
sRAGE: soluble RAGE
STAIR: Stroke Therapy Academic Industry Roundtable
TGF-β: transforming growth factor-β
TNF-α: tumor necrosis factor-α
TRANSCEND: Telmisartan Randomized Assessment Study in ACE-intolerant Subjects with Cardiovascular Disease
TrkB: tyrosine-related kinase B
VCAM-1: vascular cell adhesion molecule-1
VEGF: vascular endothelial growth factor
VSMCs: vascular smooth muscle cells
