# Correction: Kikuchi, K., *et al*., Potential of the Angiotensin Receptor Blockers (ARBs) Telmisartan, Irbesartan, and Candesartan for Inhibiting the HMGB1/RAGE Axis in Prevention and Acute Treatment of Stroke. *Int. J. Mol. Sci.* 2013, *14*, 18899–18924.

**DOI:** 10.3390/ijms15045410

**Published:** 2014-03-27

**Authors:** Kiyoshi Kikuchi, Salunya Tancharoen, Takashi Ito, Yoko Morimoto-Yamashita, Naoki Miura, Ko-ichi Kawahara, Ikuro Maruyama, Yoshinaka Murai, Eiichiro Tanaka

**Affiliations:** 1 Department of Pharmacology, Faculty of Dentistry, Mahidol University, 6 Yothe Road, Rajthevee, Bangkok 10400, Thailand; E-Mails: kikuchi_kiyoshi@kurume-u.ac.jp (K.K.); salunya.tan@mahidol.ac.th (S.T.); 2 Division of Brain Science, Department of Physiology, Kurume University School of Medicine, 67 Asahi-machi, Kurume 830-0011, Japan; E-Mail: ymurai@med.kurume-u.ac.jp; 3 Department of Neurosurgery, Kurume University School of Medicine, 67 Asahi-machi, Kurume 830-0011, Japan; 4 Department of Systems Biology in Thromboregulation, Kagoshima University Graduate School of Medical and Dental Sciences, 8-35-1 Sakuragaoka, Kagoshima 890-8520, Japan; E-Mails: takashi@m3.kufm.kagoshima-u.ac.jp (T.I.); rinken@m3.kufm.kagoshima-u.ac.jp (I.M.); 5 Department of Restorative Dentistry and Endodontology, Kagoshima University Graduate School of Medical and Dental Sciences, 8-35-1 Sakuragaoka, Kagoshima 890-8544, Japan; E-Mail: yokomaru@dent.kagoshima-u.ac.jp; 6 Laboratory of Diagnostic Imaging, Department of Veterinary Science, Faculty of Agriculture, Kagoshima University, 1-21-24 Korimoto, Kagoshima 890-0065, Japan; E-Mail: nm18@vet.kagoshima-u.ac.jp; 7 Laboratory of Functional Foods, Department of Biomedical Engineering Osaka Institute of Technology, 5-16-1 Omiya, Asahi Ward, Osaka 535-8585, Japan; E-Mail: kawahara@bme.oit.ac.jp

The original version of the paper [1] reports that “*This ACTIVE I study was supported by Pfizer*” (Page 18905). However, the sponsors of the ACTIVE I study were actually Bristol-Myers Squibb and Sanofi-Aventis rather than Pfizer. Therefore, we would like to correct the wording to read: “This ACTIVE I study was supported by Bristol-Myers Squibb and Sanofi-Aventis”. The authors apologize for any inconvenience this may have caused to the readers of this journal.
